# Estrogen receptor alpha drives mTORC1 inhibitor-induced feedback activation of PI3K/AKT in ER+ breast cancer

**DOI:** 10.18632/oncotarget.24256

**Published:** 2018-01-15

**Authors:** Wei Yang, Gary N. Schwartz, Jonathan D. Marotti, Vivian Chen, Nicole A. Traphagen, Jiang Gui, Todd W. Miller

**Affiliations:** ^1^ Department of Molecular and Systems Biology, Norris Cotton Cancer Center, Dartmouth-Hitchcock Medical Center, Geisel School of Medicine at Dartmouth, Lebanon, NH, USA; ^2^ Department of Hematology/Oncology, Norris Cotton Cancer Center, Dartmouth-Hitchcock Medical Center, Geisel School of Medicine at Dartmouth, Lebanon, NH, USA; ^3^ Department of Pathology and Laboratory Medicine, Norris Cotton Cancer Center, Dartmouth-Hitchcock Medical Center, Geisel School of Medicine at Dartmouth, Lebanon, NH, USA; ^4^ Department of Biomedical Data Sciences, Norris Cotton Cancer Center, Dartmouth-Hitchcock Medical Center, Geisel School of Medicine at Dartmouth, Lebanon, NH, USA; ^5^ Department of Comprehensive Breast Program, Norris Cotton Cancer Center, Dartmouth-Hitchcock Medical Center, Geisel School of Medicine at Dartmouth, Lebanon, NH, USA

**Keywords:** ER, mTOR, IGF-1R, feedback, everolimus

## Abstract

The mTORC1 inhibitor RAD001 (everolimus) is approved for treatment of recurrent/metastatic estrogen receptor (ER)-positive breast cancer in combination with the aromatase inhibitor (AI) exemestane. The benefits of A) continued anti-estrogen therapy for anti-estrogen-resistant disease in the context of mTORC1 inhibition, and B) adjuvant everolimus in combination with anti-estrogen therapy for early-stage disease are being tested clinically, but molecular rationale remains unclear. We hypothesized that mTORC1 inhibition activates the IGF-1R/InsR/IRS-1/2 axis in an ER-dependent manner to drive PI3K/AKT and promote cancer cell survival, implicating ER in survival signaling induced by mTORC1 inhibition. Anti-estrogen treatment synergized with RAD001 to inhibit ER+ breast cancer cell growth. Inhibition of ER, IGF-1R/InsR, or IRS-1/2 suppressed AKT activation induced by mTORC1 inhibition. RAD001 primed IGF-1R/InsR for activation, which was enhanced by ER signaling. Post-menopausal patients with early-stage ER+ breast cancer were treated presurgically +/− the AI letrozole. Viable tumor fragments from surgical specimens were treated with RAD001 and/or OSI-906 *ex vivo*; RAD001 increased AKT activation, which was abrogated by presurgical letrozole. Letrozole decreased IGF-1R and IRS-1/2 tumor levels. These data suggest that ER drives PI3K/AKT activation in response to mTORC1 inhibition, providing molecular rationale for therapeutic combinations of anti-estrogens and mTORC1 inhibitors in endocrine-sensitive disease.

## INTRODUCTION

Approximately 70% of primary breast tumors express estrogen receptor α (ER) and/or progesterone receptor (PR). Patients with such hormone receptor-positive breast cancers are typically treated with adjuvant anti-estrogen therapies such as tamoxifen or aromatase inhibitors (AIs) for 5-10 yrs after surgical removal of the primary tumor [1]. While adjuvant anti-estrogen therapies have been used to successfully treat hormone-dependent breast cancer, approximately one-third of patients develop recurrent advanced/metastatic disease that is rarely cured and frequently fatal [2, 3].

Mechanistic target of rapamycin (mTOR) complex I (mTORC1) is a critical signaling hub that regulates cellular and organismal homeostasis by balancing anabolic and catabolic processes with nutrient, energy, oxygen availability, and growth factor input signaling [4]. mTORC1 inhibitors continue to be investigated as therapeutics for many cancer subtypes. Hyperactivation of the phosphatidylinositol 3-kinase (PI3K)/AKT/mTORC1 pathway is associated with anti-estrogen resistance in ER+ breast cancer [5]. In patients with recurrent/metastatic ER+ breast cancer resistant to a non-steroidal AI (*e.g.*, letrozole, anastrozole), treatment with the steroidal AI exemestane plus the mTORC1 inhibitor everolimus (RAD001; afinitor) significantly improved median progression-free survival (but not overall survival) compared to exemestane/placebo (11 *vs*. 4.1 months) in the BOLERO-2 study [6–8]. The TAMRAD study similarly evaluated the use of tamoxifen +/− everolimus for the treatment of advanced, anti-estrogen-resistant ER+ breast cancer; patients in the combination arm experienced a prolonged time-to-progression compared to tamoxifen/placebo (8.6 *vs*. 4.5 months), and a 55% reduction in risk of death [9]. Such findings supported the U.S. FDA approval of everolimus for use in combination with exemestane for patients with ER+ breast cancer resistant to a non-steroidal AI. However, the benefit of continued anti-estrogen therapy (with exemestane) in the context of mTORC1 inhibition (with everolimus) remains unproven (being tested in the ongoing BOLERO-6 trial [10]); indeed, single-agent everolimus elicited clinical benefit in 6/22 (27%) patients with advanced ER+ breast cancer [11]. Positive findings with exemestane/everolimus in the setting of advanced disease prompted the ongoing clinical testing of adjuvant everolimus in combination with anti-estrogen therapy for patients with high-risk early-stage ER+/HER2- breast cancer [12]; many such cases will have anti-estrogen-sensitive micrometastatic disease.

Preclinical data show that combined therapeutic targeting of ER and mTORC1 is more effective than single agents to inhibit growth and induce apoptosis of breast cancer cells and xenografts [13, 14]. However, the synergistic mechanism of anti-cancer action of such drug combinations is incompletely understood. These signaling pathways exhibit crosstalk at several levels. mTORC1 and its effector ribosomal protein S6 kinase β-1 (p70S6K) catalyze serine phosphorylation of insulin receptor substrate-1 (IRS-1), inducing IRS-1 downregulation [15]. ER drives the transcription of genes encoding growth factor receptor tyrosine kinases (RTKs), ligands, and signaling adaptors, including IRS-1 and its activator, insulin-like growth factor-1 receptor (IGF-1R). Estrogen can also stimulate non-genomic ER-mediated activation of IGF-1R/insulin receptor (InsR) complexes, epidermal growth factor receptor (EGFR), Src, PI3K, and mitogen-activated protein kinase kinase (MEK) [16]. Estrogen stimulation induces ER binding to the mTORC1 component regulatory-associated protein of mTOR (Raptor), which drives nuclear translocation of Raptor. mTORC1 also phosphorylates ER on S104/106 to promote transcriptional activity [17]. We and others have shown that inhibition of mTORC1 relieves negative feedback on upstream activators, including IRS-1, IGF-1R, human epidermal growth factor receptor 3 (HER3), and PI3K, which may subsequently promote cell survival and antagonize the anti-cancer effects of mTORC1 inhibitors [18–21]. Herein, we demonstrate a role for ER in such feedback activation of survival pathways in breast cancer cell lines and human tumors, providing supportive rationale for using anti-estrogens to enhance the effects of mTORC1 inhibitors in ER+ breast cancer.

## RESULTS

### Loss of ER activity abrogates mTORC1 inhibitor-induced feedback activation of PI3K/AKT

**Figure 1:**
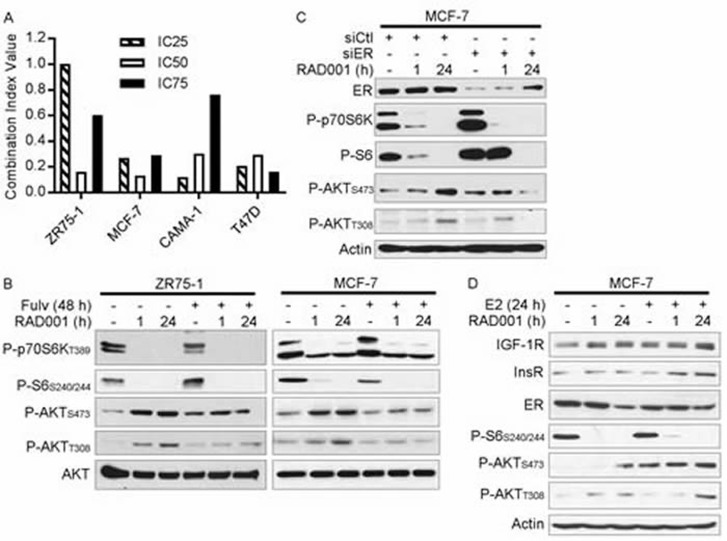
Loss of ER activity abrogates mTORC1 inhibitor-induced feedback activation of PI3K/AKT **A.** ER+ breast cancer cells were treated with dose ranges of RAD001 and/or fulv for 5 d. Relative numbers of viable cells were measured by SRB assay (full growth curves are shown in Supplementary Figure 1). Combination Index Values (CI) at the IC_25_, IC_50_, and IC_75_ for the drug combination were determined by the Median Effect method. B-D) Cell lysates were analyzed by immunoblot using the indicated antibodies. In **B.**, cells were pretreated +/− 1 μM fulv for 24 h, then treated +/− 20 nM RAD001 for an additional 1 h or 24 h (total fulv treatment time = 48 h). In **C.**, siRNA was used to knock down ER. Two days later, cells were treated +/− 20 nM RAD001 for 1 h or 24 h. In **D.**, cells were pretreated with hormone-depleted medium (10% DCC-FBS) for 3 d, then treated +/− 1 nM E2 for 24 h, 20 nM RAD001 for 1 h or 24 h, or combinations (total E2/RAD001 treatment time = 24 h).

Prior reports showed that ER+ breast cancer cells are growth-suppressed by treatment with anti-estrogens, estrogen deprivation, or mTORC1 inhibition [13, 14]. In line with these findings, we observed that treatment of 4/4 ER+ breast cancer cell lines with fulvestrant (fulv) or RAD001 suppressed growth (Supplementary Figure 1). Combined targeting of ER and mTORC1 with the combination of fulv/RAD001 synergistically inhibited growth (CI≤1 in 4/4 cell lines; Figure 1A and Supplementary Figure 1).

To explore the mechanism underlying the synergistic effects of anti-estrogens and RAD001 on growth, we pre-treated ER+ breast cancer cells +/− fulv for 24 h (to allow time for ER transcriptional effects to subside), and then co-treated +/− RAD001 for 1 h or 24 h. Treatment with RAD001 inhibited mTORC1 activity, as indicated by decreased levels of phospho-p70S6K (an mTORC1 substrate) and phospho-S6 (a p70S6K substrate) (Figure 1B). As we and others observed previously [19, 22], short-term (1 h) and longer-term (24 h) mTORC1 inhibition induced increased phosphorylation of AKT at T308 (substrate of PDK1; marker of PI3K activity) and S473 (substrate of mTORC2), implying increased activation of the PI3K/AKT pathway. Co-treatment with fulv mitigated the RAD001-induced increases in P-AKT_T308_ and P-AKT_S473_, suggesting that ER is required for mTORC1 inhibitor-induced PI3K/AKT activation in endocrine-sensitive ER+ breast cancer cells (Figure 1B and Supplementary Figure 2A/2B). The ER-specific effects of fulv were confirmed by RNAi-mediated knockdown of ER, which similarly suppressed RAD001-induced AKT phosphorylation (Figure 1C). In contrast, fulv less effectively mitigated RAD001-induced AKT activation in long-term estrogen-deprived MCF-7/LTED cells, and RAD001 increased AKT activation in fulv-resistant MCF-7/FR and T47D/FR cells despite ER inhibition with fulv (Supplementary Figure 3). Thus, combined anti-estrogen/mTORC1 inhibitor therapy may be most effective in the setting of endocrine-sensitive disease.

We then assessed whether ER activation primes the PI3K/AKT pathway for activation in response to mTORC1 inhibition. MCF-7 cells were pretreated with hormone-depleted medium (DCC-FBS) for 3 d, and then treated +/− E2 for 24 h, RAD001 for 1 h or 24 h, or combinations. Although RAD001 modestly induced P-AKT in hormone-depleted cells, the addition of E2 enhanced RAD001-induced AKT phosphorylation (Figure 1D). Thus, ligand-dependent ER signaling promotes PI3K/AKT activation in response to mTORC1 inhibition.

### mTORC1 inhibitor-induced activation of PI3K/AKT requires IGF-1R/InsR signaling

**Figure 2:**
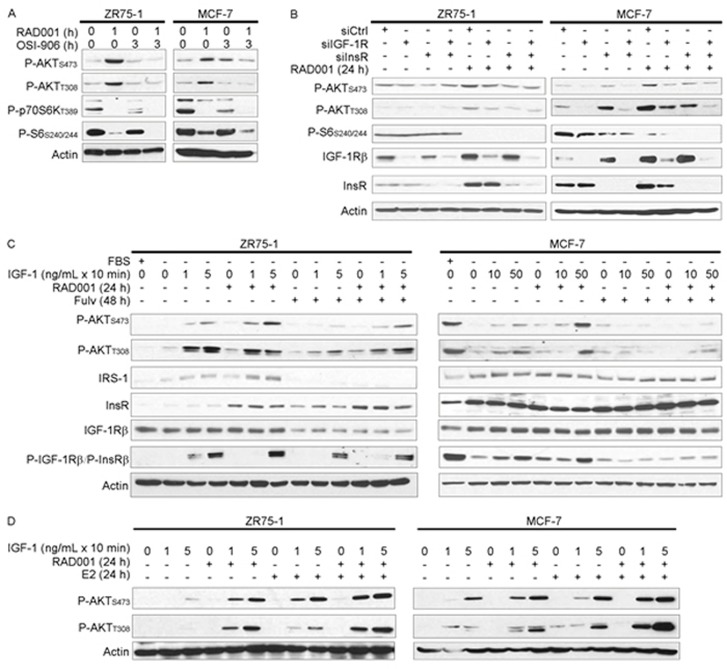
mTORC1 inhibitor-induced feedback activation of PI3K/AKT requires IGF-1R/InsR kinase activity Cell lysates were analyzed by immunoblot using the indicated antibodies. **A.** Cells were pretreated +/− 4 μM OSI-906 for 2 h, then treated +/− 20 nM RAD001 for an additional hour (total OSI-906 treatment time = 3 h). **B.** siRNA against IGF-1R, InsR, or non-silencing control was used to knock down expression. Two days post-transfection, cells were treated +/− 20 nM RAD001 for 24 h. **C.** Cells were pretreated +/− fulv (48 h) or RAD001 (24 h), with the final 24 h of pretreatment in serum-free medium as indicated. Cells were then stimulated +/− IGF-1 for 10 min. **D.** Cells were pretreated with hormone-depleted medium (DMEM/10% DCC-FBS) for 3 d, then treated +/− 20 nM RAD001 or 1 nM E2 for 24 h in serum-free medium. Cells were then stimulated with IGF-1 for 10 min.

ER promotes the transcription of genes encoding RTKs (*e.g.*, IGF-1R), ligands, and signaling adaptors (*e.g.*, IRS-1) that activate PI3K [16]. Since fulv decreased IGF-1R levels in MCF-7 and ZR75-1 cells (Supplementary Figure 2A), we considered whether this effect blunted PI3K/AKT activation in response to mTORC1 inhibition. ZR75-1 and MCF-7 cells were pretreated +/− the IGF-1R/InsR dual kinase inhibitor OSI-906 for 2 h, then treated +/− RAD001 or OSI-906 for an additional hour. OSI-906 blocked the RAD001-induced increase in P-AKT (Figure 2A). However, OSI-906 treatment partially downregulated phospho-p70S6K, suggesting partial mTORC1 inhibition that may have triggered feedback activation of AKT. To confirm the effects of OSI-906 on IGF-1R/InsR, we used RNAi to knock down IGF-1R and InsR. Dual knockdown of both RTKs blocked RAD001-induced P-AKT, while single-RTK knockdown elicited variable effects (Figure 2B), possibly due to compensation between IGF-1R and InsR [23].

We then tested the effects of mTORC1 and ER inhibition on sensitivity to IGF-1 ligand in the absence of other exogenous growth factors. Serum-deprived cells were pretreated +/− fulv or RAD001, then stimulated +/− IGF-1 for 10 min. mTORC1 inhibition with RAD001 sensitized cells to IGF-1-induced activation of AKT without appreciably changing ligand-induced phospho-IGF-1Rβ/P-InsRβ levels (Figure 2C and Supplementary Figure 4). Fulv treatment mitigated RAD001-induced AKT phosphorylation, which may have occurred by different mechanisms in different cell lines as suggested by differences between T308 and S473 phosphorylation patterns. Fulv hindered IGF-1-induced IGF-1R/InsR phosphorylation in MCF-7 cells, but had only modest effects in ZR75-1 and HCC-1428 cells (Figure 2C and Supplementary Figure 4). Serum deprivation increased total IGF-1R, InsR, and IRS-1 levels in MCF-7 cells (Figure 2C), which may reflect a loss of negative feedback regulation of expression due to decreased AKT activation [24]. In contrast, fulv decreased IRS-1 and IGF-1R levels in serum-deprived ZR75-1 cells, and IRS-1 in HCC-1428 cells (Figure 2C and Supplementary Figure 4). Thus, we postulate that fulv hindered RAD001-induced sensitization to IGF-1 in ZR75-1 and HCC-1428 cells by decreasing expression of signaling components (IRS-1, IGF-1R), and in MCF-7 cells by decreasing activation of IGF-1R/InsR; the mechanism underlying the latter is unclear.

To determine whether ER activation primes cells for response to IGF-1, cells were cultured in hormone-depleted conditions for 3 d, serum-deprived for 1 d in the presence or absence of RAD001 or E2, and then stimulated +/− IGF-1 for 10 min. Inhibition of mTORC1 or treatment with E2 each enhanced AKT activation in response to IGF-1, and combined RAD001/E2 was most effective (Figure 2D). Taken together, these data implicate ER-modulated IGF-1R/InsR signaling in mTORC1 inhibitor-induced feedback activation of PI3K/AKT.

### Requirement for IRS-1/2 in mTORC1 inhibitor-induced feedback activation of PI3K/AKT

**Figure 3:**
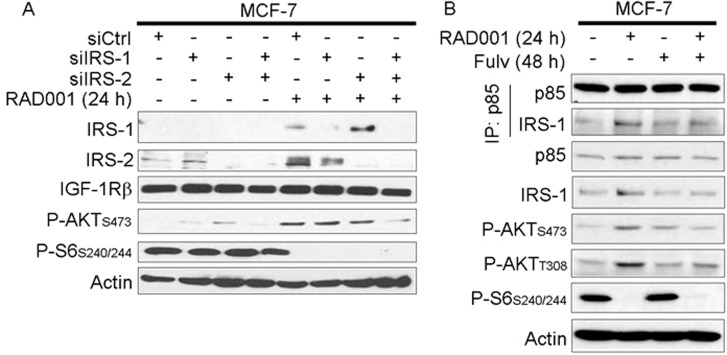
mTORC1 inhibitor-induced feedback activation of PI3K/AKT requires IRS-1/2 **A.** siRNA was used to knock down IRS-1, IRS-2, or non-silencing control. Two days later, cells were treated +/− 20 nM RAD001 for 24 h. Cell lysates were analyzed by immunoblot using the indicated antibodies. **B.** Cells were treated +/− fulv (48 h) or RAD001 (24 h), then lysates were used for immunoprecipitation of p85. Lysates and immunoprecipitates were analyzed as in (A).

IGF-1R and InsR activate PI3K by phosphorylating adaptor proteins such as IRS-1 and -2, and Tyr phosphorylated IRS-1/2 engage the p85 regulatory subunit of Class IA PI3K to promote PI3K activity [15, 25]. IRS-1/2 levels increased upon RAD001 treatment in MCF-7 cells under growth conditions (Figure 3A), which is likely due to loss of p70S6K phosphorylation that targets IRS-1/2 for proteasomal degradation [19, 26]. Using RNAi against IRS-1 and -2, we observed that dual knockdown decreased AKT activation induced by RAD001 (Figure 3A). RAD001 also increased association between the p85 regulatory subunit of PI3K and IRS-1 that was partly mitigated by fulv, possibly due to the fulv-induced decrease in IRS-1 expression (Figure 3B and Supplementary Figure 5). Thus, ER-regulated IRS-1/2 expression and interaction with PI3K are implicated in feedback induced by mTORC1 inhibition.

### Estrogen deprivation in patients with ER+ breast cancer prevents tumor AKT activation in response to mTORC1 inhibition *ex vivo*

Based on our preclinical findings, we hypothesized that inhibition of ER decreases IGF-1R/InsR/PI3K/AKT signaling induced by mTORC1 inhibition in ER+ breast cancer. To test this hypothesis in human tumors without exposing patients to an mTORC1 inhibitor, we conducted a presurgical study in which patients with Stage I-III ER+/HER2- breast cancer were treated +/− the AI letrozole for 10-21 d to induce estrogen deprivation; the 10-21-day period allows time for maximal estrogen suppression and downstream changes in the levels of ER-regulated transcripts [27]. At the time of surgical resection of the tumor, viable tumor fragments were acquired, adapted to serum-free medium *ex vivo* for 5 h, then treated +/− RAD001 or OSI-906 for 1 h. Tumor lysates were analyzed by immunoblot to assess AKT phosphorylation.

**Figure 4:**
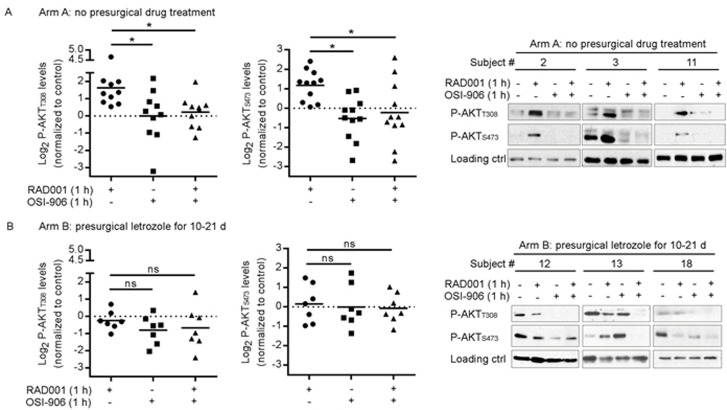
Presurgical estrogen deprivation in patients with ER+ breast cancer prevents RAD001-induced PI3K/AKT activation in tumors *ex vivo* Patients with early-stage ER+/HER2- breast cancer received either **A.** no presurgical treatment (Arm A, *n* = 10), or **B.** presurgical treatment with letrozole for 10-21 d (Arm B, *n* = 7). Within 1 h after surgical resection, 1-mm^3^ punch cores were taken from primary tumors *ex vivo*. Tumor cores were put in serum-free medium for 5 h, then treated +/− 20 nM RAD001, 4 μM OSI-906, or the combination for 1 h. Tumor lysates were analyzed by immunoblot using the indicated antibodies. Immunoblot film signals were quantified by densitometry. Signal values from control-treated tumors were set at “1.” Data are shown as log_2_ P-AKT of inhibitor-treated sample relative to control. Horizontal bars indicate mean values. *p < 0.05 by Bonferroni multiple comparison-adjusted post-hoc test. **C.** Representative results are shown at right from 3 patients’ tumors from Arms A and B; actin or vinculin was assessed to confirm equal loading.

We first evaluated tumors from 10 patents in Arm A who did not receive presurgical anti-cancer treatment. *Ex vivo* treatment of tumor fragments with RAD001 increased the levels of AKT phosphorylation on both T308 and S473 (Figure 4A). When quantifying (phospho-)protein levels by densitometry (control-treated tumors were set at “1”), we observed a 45% to 2,400% increase in P-AKT_T308_ compared to control (mean % change +/− SD = 388% +/− 623%), and up to a 431% increase in P-AKT_S473_ (mean % change +/− SD = 157% +/− 35%). Treatment with OSI-906 alone or in combination with RAD001 significantly decreased P-AKT levels compared to single-agent RAD001 (Figure 4A), confirming our *in vitro* findings (Figure 2A). These results suggest that mTORC1 inhibition induces PI3K/AKT activation in an IGF-1R/InsR kinase-dependent manner in human ER+ breast tumors.

We then analyzed tumors from 7 patents in Arm B who received 10-21 d of letrozole treatment prior to surgery. *Ex vivo* treatment of Arm B tumors with RAD001 did not significantly increase P-AKT levels: P-AKT_T308_ ranged from −51% to 163% compared to control (mean % change +/− SD = −10% +/− 38%), and P-AKT_S473_ measured −51% to 281% compared to control (mean +/− SD = 135% +/− 91%). Accordingly, OSI-906/RAD001 co-treatment did not significantly alter P-AKT levels compared to RAD001 alone (Figure 4B). These data suggest that estrogen-induced ER activation is required for mTORC1 inhibitor-induced activation of PI3K/AKT in human ER+ breast tumors.

**Figure 5:**
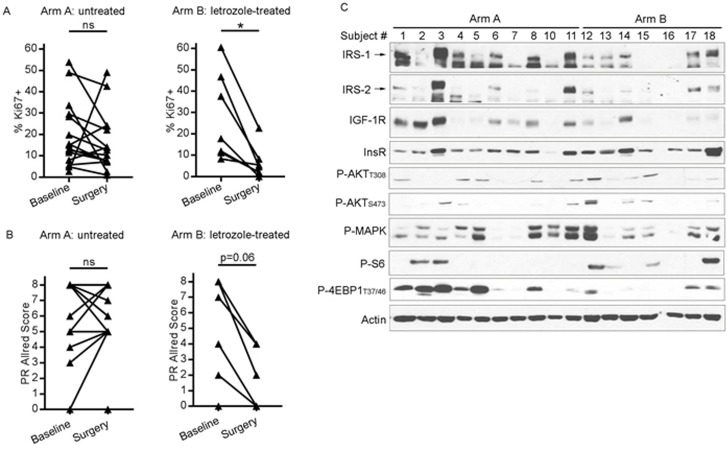
Presurgical estrogen deprivation in patients with ER+ breast cancer decreases tumor cell proliferation and IGF-1R/IRS-1/IRS-2 expression A/B) Formalin-fixed, paraffin-embedded diagnostic tumor biopsies (baseline) and surgical specimens [post-letrozole (Arm B) or untreated (Arm A)] were analyzed by IHC using antibodies against Ki67 **A.** or PR **B.** *p<0.05 by Wilcoxon test. ns- not significant. **C.** Lysates from frozen surgical tumor specimens were analyzed by immunoblot using the indicated antibodies.

Presurgical anti-estrogen treatment often suppresses cell proliferation in ER+ breast tumors [28]. To confirm the growth-suppressive effects of presurgical letrozole, we measured tumor cell proliferation by Ki67 IHC. Tumor Ki67 scores were not significantly different between baseline biopsies and surgical specimens from patients who did not receive presurgical treatment (Arm A). In contrast, presurgical letrozole significantly decreased Ki67 score in Arm B (Figure 5A and Supplementary Figure 6). Presurgical letrozole also induced a trend towards decreased tumor PR levels (*p* = 0.06), reflecting reduced ER transcriptional activity, while tumors from untreated patients showed no notable difference between baseline and surgical specimens (Figure 5B and Supplementary Figure 6). Letrozole did not appreciably alter ER expression (Supplementary Figures 6-7).

We further evaluated surgical tumor specimens by immunoblot analysis of lysates. Post-letrozole tumors showed lower IGF-1R, IRS-1, and IRS-2 levels than tumors from untreated patients (Figure 5C); similar differences were detected by IGF-1R IHC (Supplementary Figures 6-7). Letrozole-treated tumors also showed decreased phosphorylation of 4EBP1 and MAPK compared to untreated tumors (Figure 5C), suggesting that estrogen deprivation suppresses mTORC1 and MEK activation. These findings from human tumors collectively suggest that 1) ER drives the expression of IGF-1R, IRS-1, and IRS-2, and 2) IGF-1R/InsR kinase activity is required for feedback activation of PI3K/AKT upon inhibition of mTORC1, providing mechanistic insight into the effects of mTORC1 and ER inhibition on signaling in ER+ breast cancer.

## DISCUSSION

**Figure 6:**
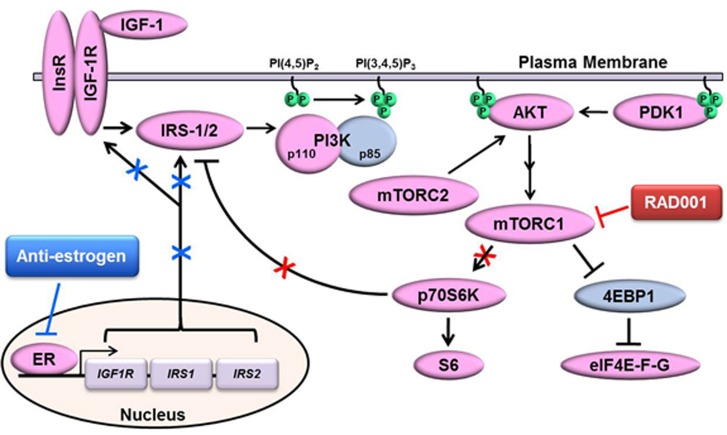
Proposed model of ER-mediated control of mTORC1 inhibitor-induced activation of PI3K/AKT Depicted is the canonical signaling pathway in which ligand-activated IGF-1R/InsR homo- and hetero-dimers phosphorylate IRS-1/2 at Tyr sites, creating docking sites for p85/PI3K that derepress p110/PI3K. p110/PI3K converts PIP_2_ to PIP_3_, and PIP_3_ enables recruitment of PH domain-containing proteins (*e.g.*, AKT, PDK1) to the cytoplasmic face of the plasma membrane. PDK1 and mTORC2 phosphorylate AKT, and activated AKT in turn signals through the TSC1/2 complex to activate mTORC1. mTORC1 and its phospho-activated substrate p70S6K phosphorylate IRS-1/2 at Ser residues to promote proteasomal degradation of IRS-1/2, creating a negative feedback loop. Inhibition of mTORC1 with RAD001 blocks such negative feedback, leading to increased IRS-1/2/PI3K/AKT activation. ER drives the expression of IGF-1R and IRS-1/2, which are downregulated by anti-estrogen treatment. Thus, anti-estrogens mitigate mTORC1 inhibitor-induced activation of PI3K/AKT in ER+ breast cancer cells.

Herein, we provide evidence from ER+ breast cancer cell lines and human tumors implicating ER in signaling responses to mTORC1 inhibition. mTORC1 inhibitors generally have a cytostatic effect on ER+ breast cancer cells [13, 14], which is thought to be due in part to a loss of negative feedback signaling to activators upstream of PI3K including RTKs and IRS-1 [18–21], driving activation of PI3K/AKT to promote cell survival. We demonstrated that mTORC1 inhibition with RAD001 (everolimus) induces IGF-1R/InsR/IRS-1/IRS-2-dependent activation of PI3K/AKT signaling in ER+ breast cancer cells and human tumors treated *ex vivo*. RAD001 synergized with the anti-estrogen fulv to inhibit growth in 4/4 ER+ breast cancer cell lines. Inhibition of ER with fulv, RNAi (*in vitro*), or estrogen deprivation (AI treatment of patients) decreased mTORC1 inhibitor-induced PI3K/AKT activation in endocrine-sensitive cells and tumors. In most cases, anti-estrogen treatments decreased expression of IGF-1R/IRS-1/IRS-2 signaling components, which may be the predominant mechanism by which ER drives such feedback signaling (Figure 6). However, it is also possible that ER-mediated non-genomic signaling, such as *via* IGF-1R/InsR [22, 29], drives PI3K/AKT activation in response to mTORC1 inhibition.

The extensive crosstalk between the ER and PI3K/AKT/mTORC1 pathways provides rationale to target these pathways in ER+ breast cancer. However, there is a lack of understanding of the reciprocity of this crosstalk. There are numerous examples of PI3K/AKT/mTORC1 signaling regulating ER activity, including AKT-, mTOR-, and p70S6K-mediated phosphorylation and activation of ER, and AKT-mediated phosphorylation of c-Jun, which complexes with ER [30–36]. However, the mechanism(s) by which ER affects PI3K/AKT/mTORC1 signaling remain to be fully elucidated. In a positive feedback model, ER promotes the transcription of genes encoding RTKs, ligands, and signaling adaptors, which are predicted to increase PI3K pathway activation. Anti-estrogen treatment downregulated IGF-1R, IRS-1, and IRS-2 levels in ER+ breast cancer cells and human tumors (Figures 2C, 5C, Supplementary Figure 2A, Supplementary Figure 4, Supplementary Figure 5, Supplementary Figure 7C/7D), although effects varied between biological systems. Accordingly, clinical evidence suggests that ER function sustains PI3K activation. In patients with ER+ breast cancer, 6 months of neoadjuvant treatment with letrozole +/− cyclophosphamide reduces P-AKT_S473_ and P-mTOR_S2448_ tumor levels, which correlate with improved response and disease outcome [37]. In a second study, 14 d of neoadjuvant letrozole modestly decreased P-S6 levels [38]. Similarly, we showed that presurgical letrozole decreased P-4EBP1 levels (Figure 5C). Hence, anti-estrogens may suppress ER+ breast cancer growth in part by decreasing PI3K/AKT/mTOR signaling. The findings presented herein closely align with a positive feedback model, and mechanistically show how blocking ER can abrogate activation of PI3K/AKT in response to mTORC1 inhibition.

Clinical and preclinical data also support a negative feedback model of ER-PI3K/AKT/mTORC1 crosstalk in which these pathways antagonize each other. ER and PR levels are inversely correlated with markers of PI3K/AKT/mTOR activation in primary ER+ breast tumors [5, 39]. Adaptation to long-term ER inhibition (with fulv or hormone deprivation) increases PI3K/AKT/mTORC1 activity in ER+ breast cancer cells [5, 40–42]. PI3K inhibitor treatment of patients with ER+ breast cancer for ≥14 d induces upregulation of tumor ER levels and activity [43], which Toska *et al*. recently demonstrated is mediated by loss of AKT-induced epigenetic silencing of ER activity [44]. Furthermore, we previously showed that adaptation of ER+ breast cancer cells to long-term PI3K inhibition induces upregulation of ER and PR [45]. Thus, substantial data support seemingly conflicting models of positive and negative feedback between the ER and PI3K/AKT/mTORC1 pathways, resolution of which will require further study.

AKT phosphorylation at both T308 and S473 is required for maximal kinase activation [46]. Phosphorylation of T308 is mediated by the PI3K effector PDK1, while S473 phosphorylation is mediated by mTORC2 [47, 48]. The effects of mTORC1 inhibition on AKT phosphorylation varied between cell lines: RAD001 drastically increased both P-AKT_T308_ and P-AKT_S473_ in ZR75-1 and MCF-7 cells, but only robustly increased P-AKT_S473_ in HCC-1428 cells (Figure 1B and Supplementary Figure 2). These observations indicate cell line-specific differences in PI3K-PDK1-mTORC2-AKT programming, and suggest that inhibition of ER and mTORC1 affect different feedback circuits upstream of T308 and/or S473. AKT has 3 human isoforms (AKT1, AKT2, and AKT3) with overlapping and distinct biological functions [46], and similar amino acid motifs surrounding T308 and S473. Since phospho-AKT antibodies do not distinguish between isoforms, it is possible that isoform-specific differences in AKT phosphorylation contributed to the observed differences between cell lines.

In 2012, the U.S. FDA approved everolimus (RAD001) as the first PI3K/AKT/mTOR pathway inhibitor for the treatment of advanced ER+ breast cancer in combination with the steroidal AI exemestane following progression on a non-steroidal AI. The BOLERO-2 study showed that combined exemestane/everolimus therapy increased median PFS from 4.1 to 10.6 months compared to exemestane/placebo [6]. An exploratory subgroup analysis revealed that patients with cancer that had progressed during or within 12 months after the end of (neo)adjuvant AI therapy, rendering exemestane +/− everolimus the first-line therapy for their advanced disease, experienced even greater benefit from the addition of everolimus: exemestane/everolimus increased median PFS to 15.2 months from 4.2 months with exemestane/placebo [49]. Lingering questions remain regarding 1) the optimal time in the disease course to introduce everolimus, 2) the need for continued anti-estrogen therapy for anti-estrogen-resistant disease in the context of mTORC1 inhibition with everolimus, and 3) the clinical significance of mTORC1 inhibitor-induced feedback activation of upstream pathways (*i.e.*, RTKs, PI3K, AKT). Herein, we provide evidence from preclinical models and functional human tumor studies demonstrating a role for ER in RAD001-induced PI3K/AKT activation in endocrine-sensitive ER+ breast cancer. These findings provide rationale for anti-estrogen co-treatment in the setting of endocrine-sensitive ER+ disease being treated with an mTORC1 inhibitor; such drug combinations are currently being tested clinically in the adjuvant setting [12].

## MATERIALS AND METHODS

### Cell lines and RNA interference

Parental cell lines were obtained from ATCC and cultured in DMEM (Gibco) with 10% FBS (Hyclone) for <3 months before analysis. Long-term estrogen-deprived (LTED) cells were generated through culture in phenol red-free DMEM with 10% dextran-charcoal-treated FBS (DCC-FBS) for 3 months. Cells resistant to the anti-estrogen fulvestrant (fulv; MCF-7/FR, T47D/FR) were gifts from Matthew Ellis (Washington University), and maintained in DMEM/10% FBS with 1 μM fulv. LTED and FR cells were authenticated by STR genotyping (Univ. of Vermont Advanced Genome Technologies Core). Cells were transfected with siRNA targeting IGF-1R (#GS3480), InsR (#GS3643), IRS-1 (#GS3667), IRS-2 (#GS8660), or non-silencing control (#1027310) from Qiagen using Lipofectamine RNAiMAX per manufacturer’s instructions (Life Technologies). Cells were treated with phenol red-free DMEM containing 10% dextran-charcoal-treated FBS (DCC-FBS; Hyclone), RAD001, OSI-906 (Selleck Chemicals), fulv (Tocris Bioscience), IGF-1 (R&D Systems), or 17β-estradiol (E2; Sigma) as indicated.

### Sulforhodamine B (SRB) assay

Cells were seeded in triplicate at 3,000-5,000/well in 96-well plates. The following day, cells were treated as indicated for 5 d. Relative numbers of adherent cells were determined by SRB staining [50]. IC_50_ values were determined using Calcusyn software (Biosoft), where the mean Abs_490_ value of triplicate wells was used. For drug synergy studies, cells were treated with fulv and RAD001 at fixed ratios of IC_50_ values. The combination indices (CI) for the drug combination at the IC_25_, IC_50_, and IC_75_ were determined by the Median Effect method [51] using Calcusyn software. CI<1 is indicative of synergy.

### Patients and *ex vivo* tumor culture

Seventeen patients with stage I-III invasive ER+/HER2- breast cancer were recruited to clinical study NCT02010021. The clinical study protocol was approved by the Dartmouth College Institutional Review Board. All patients provided signed informed consent. This study was conducted in accordance with good clinical practice and all applicable regulatory requirements, including the 1996 version of the Declaration of Helsinki. Tumors were required to have ≥50% ER+ cancer cells, and be HER2- [immunohistochemistry (IHC) 0-1+, or with a FISH ratio of <1.8 if IHC is 2+ or if IHC had not been done]. The first 10 patients received no presurgical anti-cancer therapy (Arm A), and an interim analysis was performed. The next 7 patients were presurgically treated with the AI letrozole for 10-21 d (Arm B). Primary tumors were surgically resected as standard of care, and tumor fragments for research use were then dissected from the surgical specimen within 1.5 h of removal from the patient. Tumor fragments were transported in serum-free DMEM on ice. Using a 1-mm biopsy punch device (Miltex), ~40 viable tumor cores were dissected from each tumor specimen within 30 min. Tumor cores (~10 per treatment group) were cultured *ex vivo* in serum-free DMEM for 5 h, then treated +/− 20 nM RAD001, 4 μM OSI-906, or the combination for 1 h. Tumor cores were then snap-frozen, lysed, and analyzed by immunoblot.

### Immunoprecipitation and immunoblotting

Immunoblotting of lysates from cells and frozen tumor fragments was performed as described [52]. Immunoprecipitation of p85 from cell lysates was performed using Dynal protein-G beads (Invitrogen) and p85 antibody (Abcam) as described [53]. Following SDS-PAGE, proteins were transferred to nitrocellulose membranes, and even protein loading was visually confirmed by Ponceau S staining. Blots were probed with antibodies against P-AKT_S473_, P-AKT_T308_, AKT, P-p70S6K_T389_, P-S6_S240/244_, P-IGF-1Rβ_Y1135/6_/P-InsRβ_Y1150/1_, IGF-1Rβ, InsRβ, IRS-1, IRS-2, actin, vinculin (Cell Signaling), p85 (Abcam), or ER (Santa Cruz). HRP-labeled secondary antibodies (GE Healthcare) and ECL substrate (Pierce) were used for signal detection. Densitometry analysis of immunoblot film was performed using ImageJ software, and relative signal values were analyzed by ANOVA followed by Bonferroni multiple comparison-adjusted post-hoc test between groups.

### Immunohistochemistry (IHC)

Five-micron sections of formalin-fixed, paraffin-embedded (FFPE) tumor tissues acquired from patients at baseline (diagnostic biopsy) and at the time of surgery (untreated or after presurgical letrozole) were used for IHC staining with antibodies against Ki67 (Biocare Medical), ER (Cell Marque, SP1), PR (Leica Biosystems, # NCL-l-PGR-312), or IGF-1R (Cell Signaling, #3027). IHC evaluation was performed by a breast pathologist blinded to treatment arm; details are provided in Supplementary Information. In Arm A, two patients had bilateral tumors, and two patients had two ipsilateral tumors; all were analyzed by IHC.

## SUPPLEMENTARY MATERIALS FIGURES



## Abbreviations

ER: estrogen receptor alpha
HER3: human epidermal growth factor receptor 3
IGF-1R: insulin-like growth factor-1 receptor
IHC: immunohistochemistry
InsR: insulin receptor
IRS-1: insulin receptor substrate-1
IRS-1: insulin receptor substrate-2
MAPK: mitogen-activated protein kinase (also called ERK1/2)
MEK: mitogen-activated protein kinase kinase
mTOR: mechanistic target of rapamycin
mTORC1: mTOR complex I
p70S6K: ribosomal protein S6 kinase beta-1
PI3K: phosphatidylinositol 3-kinase
PFS: progression-free survival
PR: progesterone receptor
Raptor: regulatory-associated protein of mTOR
RTK: receptor tyrosine kinase
SRB: Sulforhodamine B
