# Supplementary material for: Estrogen receptor alpha drives mTORC1 inhibitor-induced feedback activation of PI3K/AKT in ER+ breast cancer
Source: Oncotarget. 2018 Jan 15;9(10):8810–22. doi: 10.18632/oncotarget.24256 (PMC5823630; doi:10.18632/oncotarget.24256)
Supplement: Supplementary file 1 [file oncotarget-09-8810-s001.pdf]

Estrogen receptor alpha drives mTORC1 inhibitor-induced feedback activation of PI3K/AKT in ER+ breast cancer

SUPPLEMENTARY MATERIALS AND METHODS

Immunohistochemistry (IHC)

ER and PR expression levels were manually evaluated using the Allred scoring method. The proportion of stained cells was divided into 6 categories (0=0%; 1=<1%; 2=1-10%; 3=11-33%; 4=34-66%; 5=67-100%) and intensity was assigned as 0-3 (0=negative; 1=weak; 2=intermediate; 3=strong). The proportion and intensity scores were added to generate an Allred Score of 0-8 [1]. Whole slides were scanned at 40x (Aperio AT2, Leica Biosystems), and automated Ki67 analysis (percent positive nuclei) was determined using the Aperio ImageScope (v12.3.1.60002, Leica Biosystems) nuclear v9 algorithm. As recommended by the International Ki67 in Breast Cancer Working Group, 3 high-power microscopic fields were selected for analysis to represent the spectrum of staining present on the whole tissue section, and a minimum of 500 malignant invasive cells were scored [2]. A mean percentage of Ki67 positivity was determined for each tumor specimen. IGF-1R immunostaining was manually evaluated using a previously reported scoring system [3]. Membranous IGF-1R expression was scored as 0-3+ (0=negative; 1+=incomplete staining in >10% tumor cells; 2+=weak or moderate complete staining in >10% tumor cells; 3+=strong complete staining in >10% tumor cells). Tumors were considered negative for IGF-1R if 0 or 1+, and positive if 2+ or 3+.

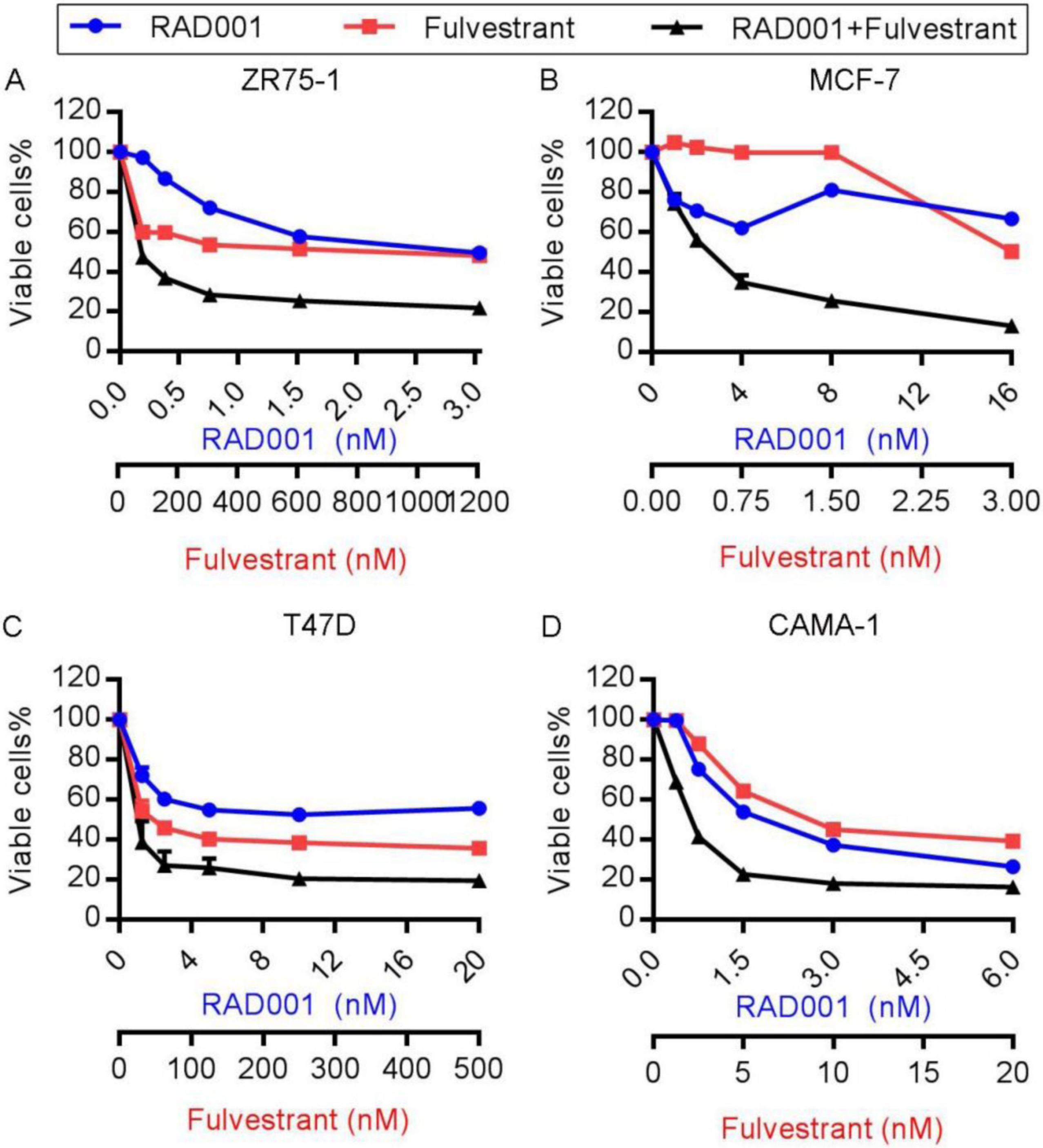

Supplementary Figure 1: RAD001 synergizes with fulvestrant to inhibit cell growth. ER+ breast cancer cells were treated with dose ranges of RAD001 and/or Fulvestrant at fixed ratios of IC_{50} values for 5 d. Relative numbers of viable cells were measured by SRB assay. These data were used to calculate Combination Index Values presented in Figure 1A.

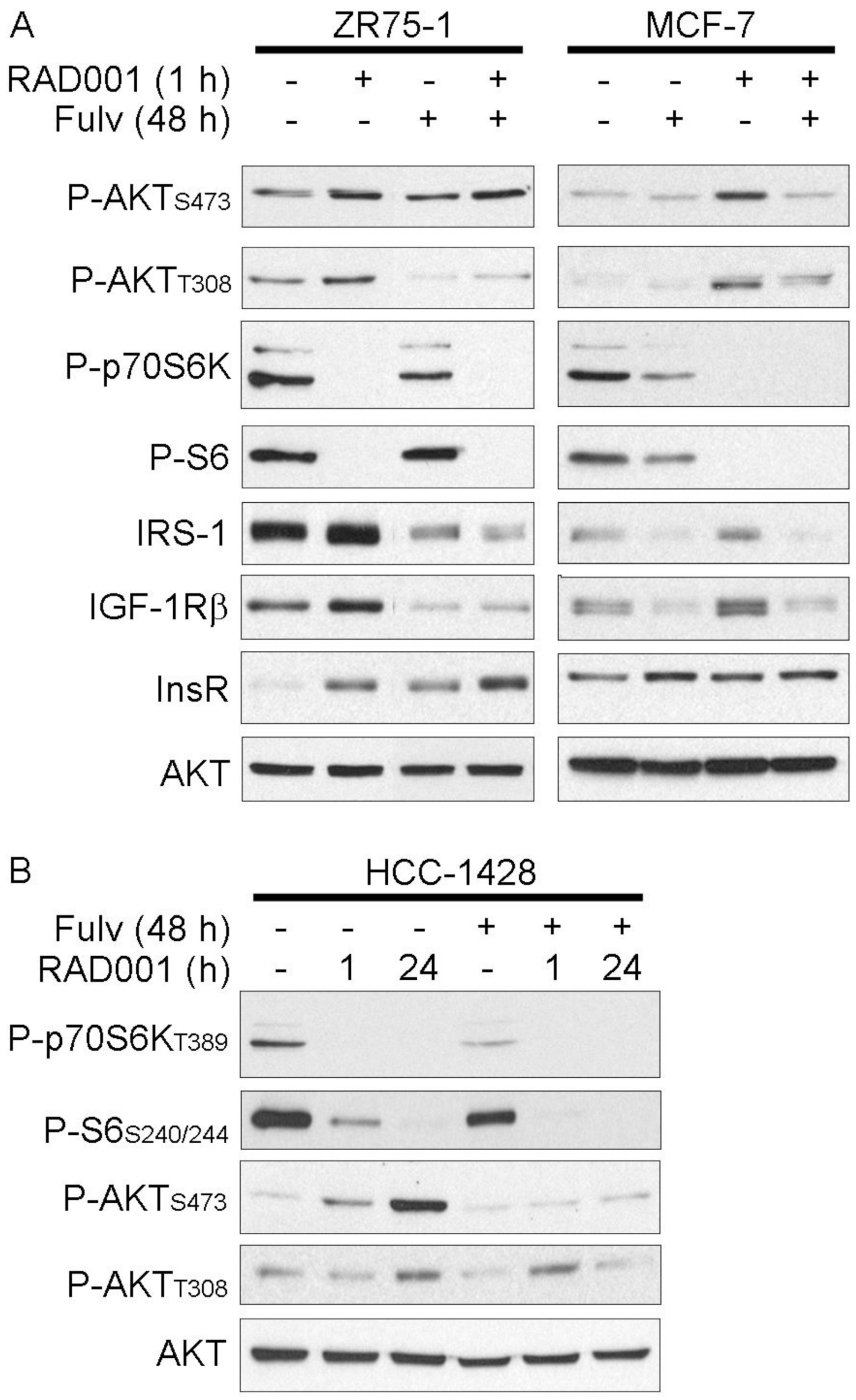

Supplementary Figure 2: ER signaling regulates mTORC1-induced PI3K/AKT activation. Cell lysates were analyzed by immunoblot using the indicated antibodies. **A.** Cells were pretreated +/- 1 µM fulv for 48 h, then co-treated +/- 20 nM RAD001 for 1 h. **B.** Cells were pretreated +/- 1 µM fulv for 24 h, then co-treated +/- 20 nM RAD001 for 1 h or 24 h (total fulv treatment time = 48 h).

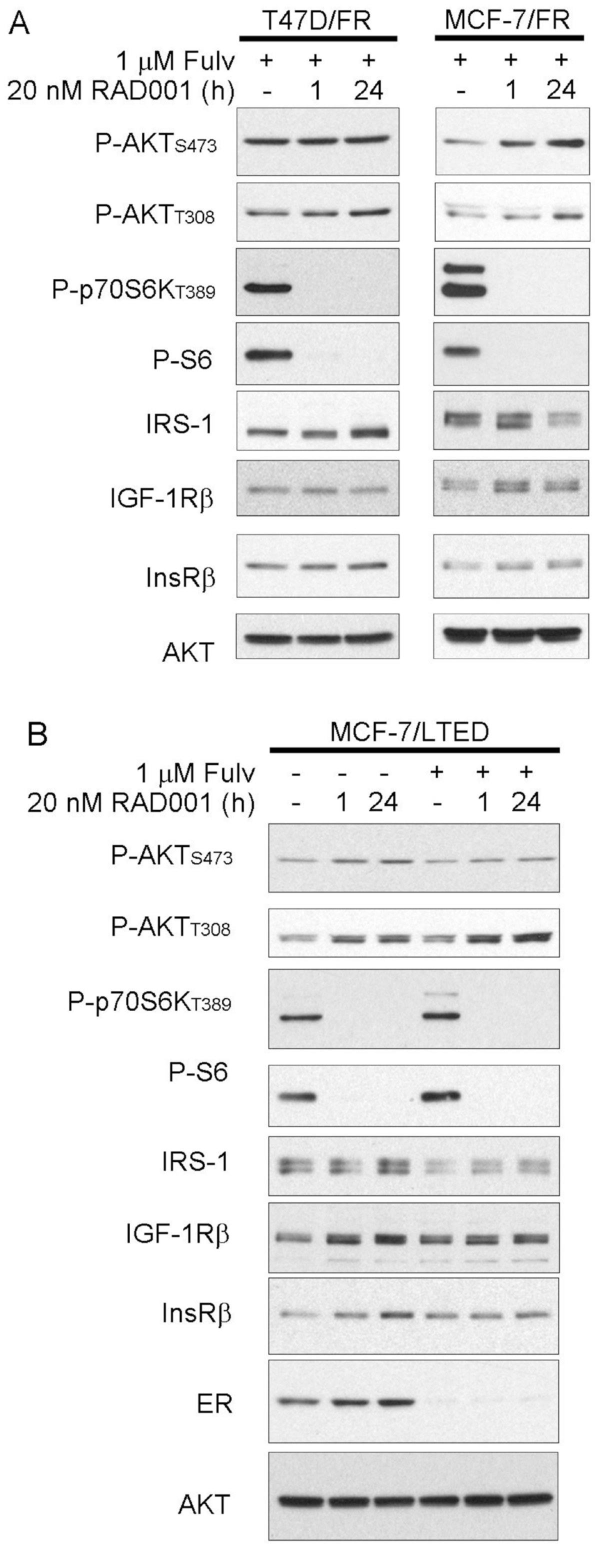

Supplementary Figure 3: ER+ breast cancer cells with acquired endocrine resistance retain the ability to activate PI3K/AKT in response to mTORC1 inhibition. Cell lysates were analyzed by immunoblot using the indicated antibodies. **A.** Fulv-resistant (FR) cells maintained in 1 µM fulv were treated +/- 20 nM RAD001 for 1 h or 24 h. **B.** Long-term estrogen-deprived (LTED) cells maintained in hormone-depleted medium (with 10% DCC-FBS) were pretreated +/- 1 µM fulv for 24 h, then co-treated +/- 20 nM RAD001 for 1 h or 24 h (total fulv treatment time = 48 h).

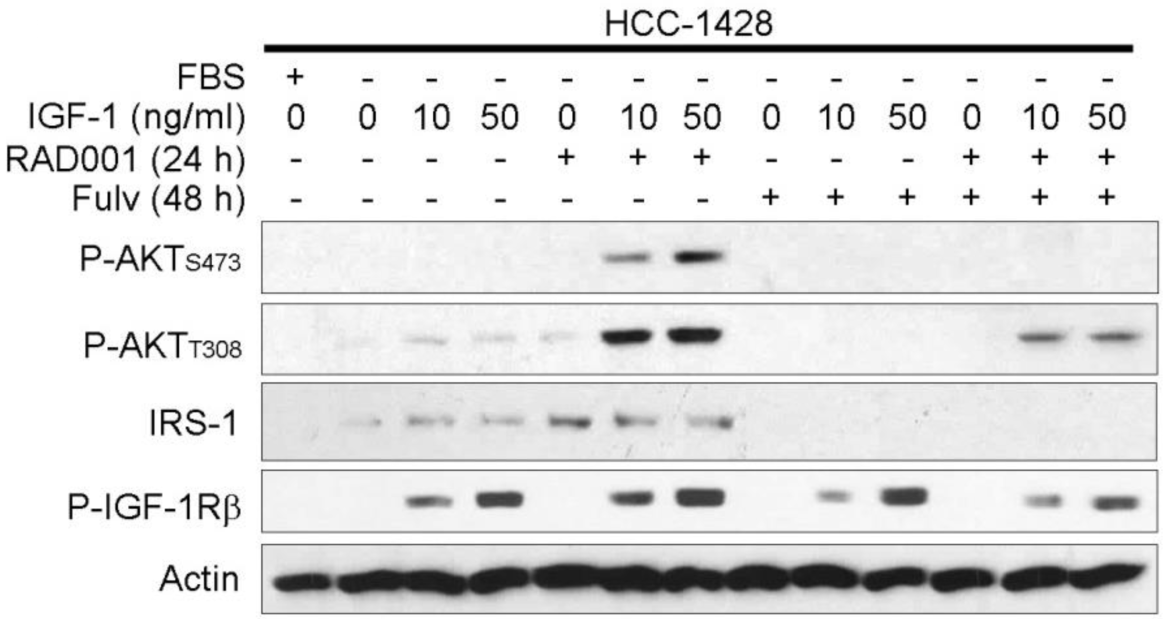

Supplementary Figure 4: mTORC1 inhibitor-induced feedback activation of PI3K/AKT requires IGF-1R/InsR signaling. HCC-1428 cells were pretreated +/- 1 μM fulv for 24 h, then deprived of serum +/- 20 nM RAD001 or fulv for the next 24 h. Cells were then stimulated +/- IGF-1 for 10 min. Lysates were analyzed by immunoblot using the indicated antibodies. Lane 1 reflects lysate from cells maintained in growth medium (DMEM/10% FBS).

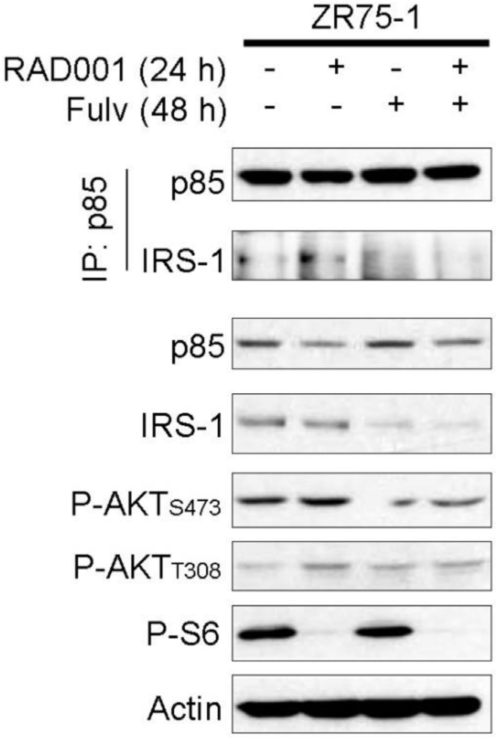

Supplementary Figure 5: mTORC1 inhibitor-induced feedback activation of PI3K/AKT requires IRS-1/2. Cells were pretreated +/- 1 μM fulv for 24 h, then co-treated +/- 20 nM RAD001 for 24 h (total fulv treatment time = 48 h). Cell lysates were used for immunoprecipitation of p85. Immunoprecipitates and lysates were analyzed by immunoblot using the indicated antibodies.

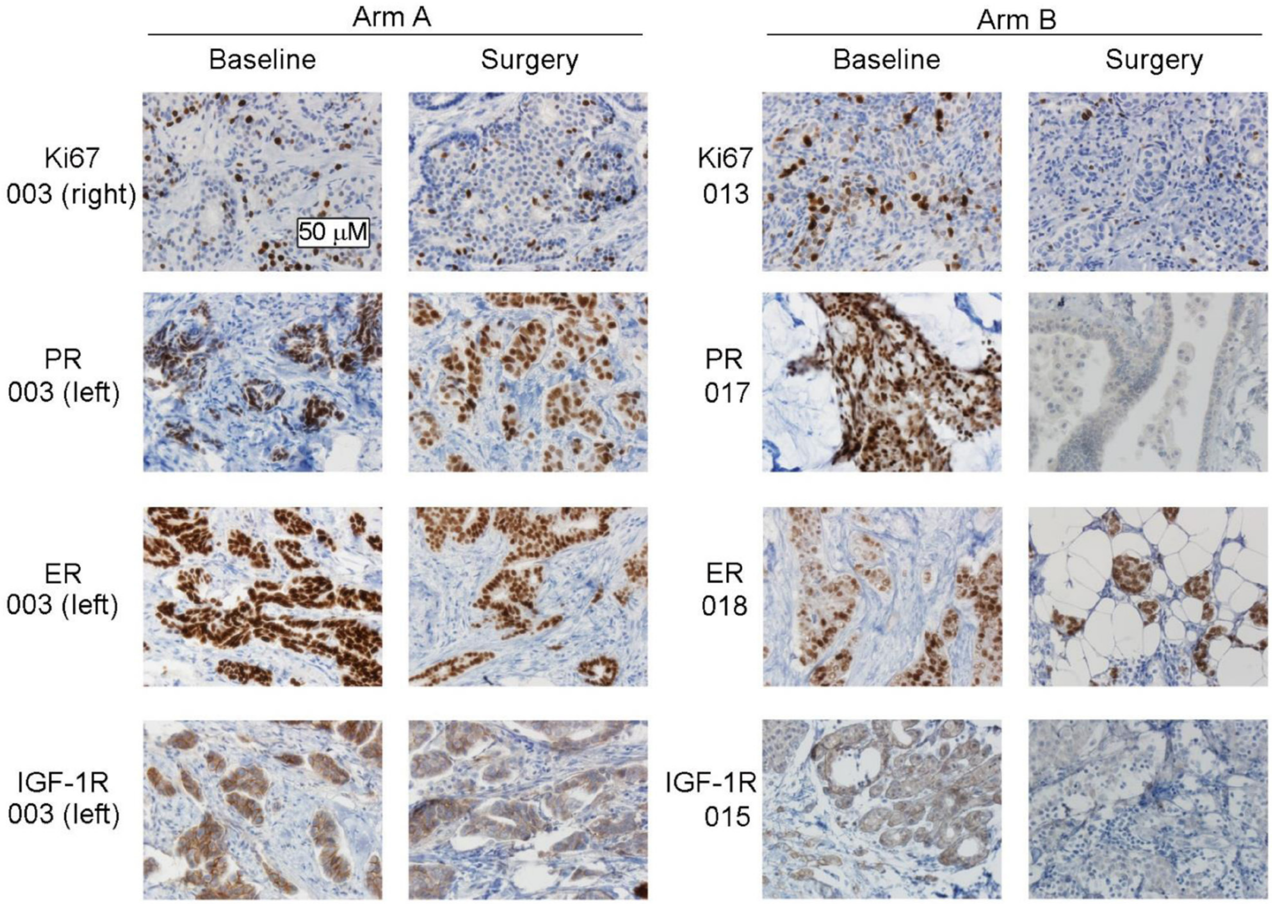

Supplementary Figure 6: Presurgical estrogen deprivation decreases tumor cell proliferation and PR expression in patients with ER+ breast cancer. Patients with early-stage ER+/HER2- breast cancer received either **A.** no presurgical treatment (Arm A, *n*=10), or **B.** presurgical treatment with letrozole for 10-21 d (Arm B, *n*=7). Sections of baseline (diagnostic biopsy) and sugical tumor specimens were analyzed by IHC using antibodies against Ki67, PR, ER, and IGF-1R. Representative IHC images are shown.

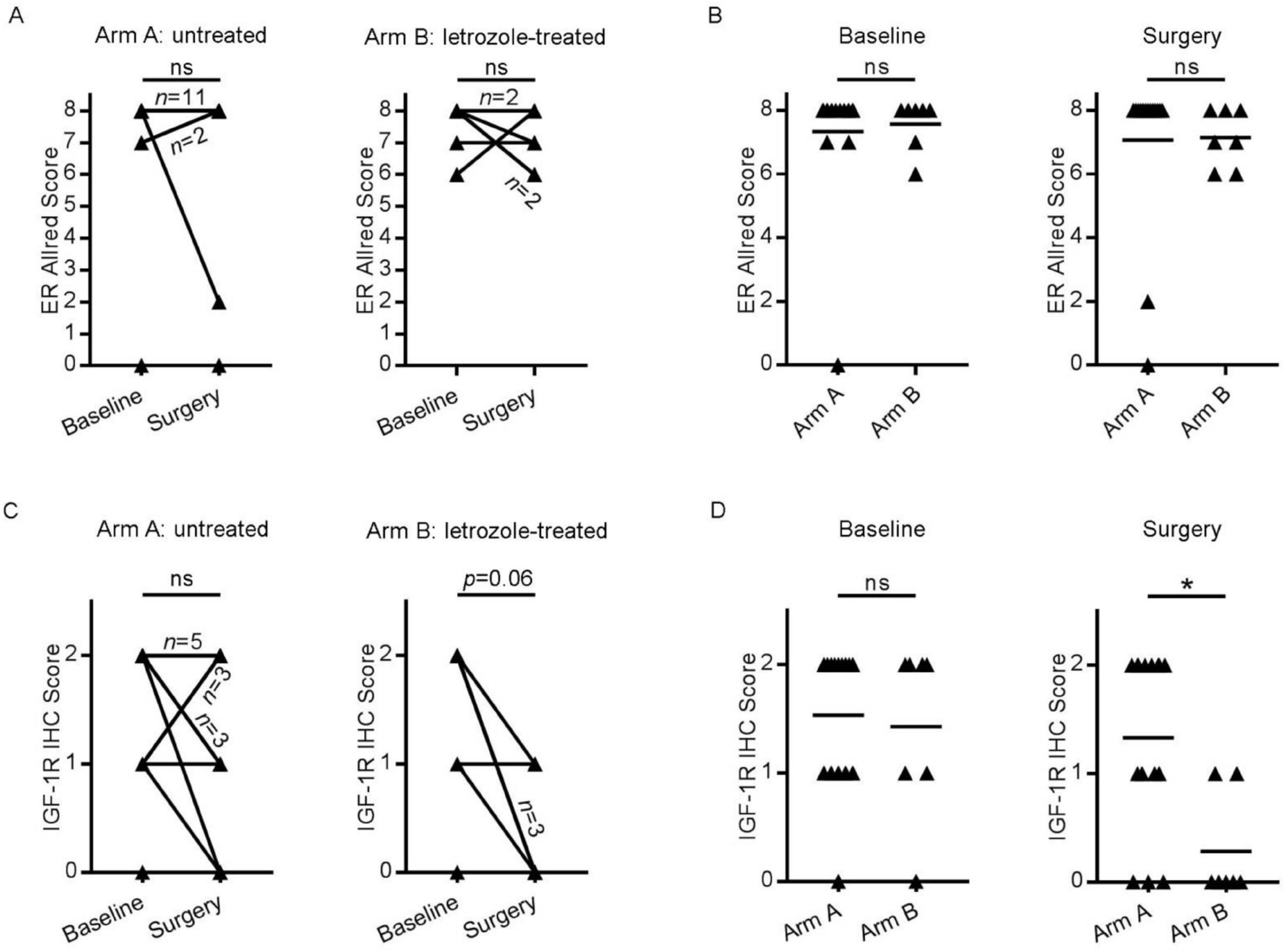

Supplementary Figure 7: Presurgical estrogen deprivation decreases tumor IGF-1R expression in patients with ER+ breast cancer. Patients with early-stage ER+/HER2- breast cancer received either **A.** no presurgical treatment (Arm A, *n*=10), or **B.** presurgical treatment with letrozole for 10-21 d (Arm B, *n*=7). Sections of baseline (diagnostic biopsy) and sugical tumor specimens were analyzed by IHC using antibodies against ER and IGF-1R. A/B) ER Allred scores comparing matching baseline and surgical tumor specimens separated by treatment arm (A), and comparing tumor specimens between treatment arms at each time point (B). **C./D.** IGF-1R IHC scores comparing matching baseline and surgical tumor specimens separated by treatment arm (C), and comparing tumor specimens between treatment arms at each time point (D). In (A) and (C), IHC scores were compared by Wilcoxon test. In (B) and (D), IHC scores were compared by Mann-Whitney U-test. ns- not significant.

SUPPLEMENTARY REFERENCES

1. Allred DC, Harvey JM, Berardo M, Clark GM. Prognostic and predictive factors in breast cancer by immunohistochemical analysis. Mod Pathol. 1988; 11: 155-68.
2. Dowsett M, Lonning PE. 10th International Aromatase Conference Proceedings, September 2010. Preface. Steroids. 2011; 76: 729.
3. de Groot S, Charehbili A, van Laarhoven HW, Mooyaart AL, Dekker-Ensink NG, van de Ven S, Janssen LG, Swen JJ, Smit VT, Heijns JB, Kessels LW, van der Straaten T, Bohringer S, et al. Insulin-like growth factor 1 receptor expression and IGF1R 3129G > T polymorphism are associated with response to neoadjuvant chemotherapy in breast cancer patients: results from the NEOZOTAC trial (BOOG 2010-01). Breast Cancer Res. 2016; 18: 3.
